# 628. A meta-analysis of Tick Paralysis in humans globally

**DOI:** 10.1093/ofid/ofad500.694

**Published:** 2023-11-27

**Authors:** Sarah N Matthews, Chawki Harfouch

**Affiliations:** Loma Linda University Medical Center, Murrieta, California; Loma Linda University Medical Center, Murrieta, California

## Abstract

**Background:**

Tick paralysis is a neurological disease that causes acute ascending motor paralysis and sparing of sensory function. It is caused by a neurotoxin released from the tick during hematophagy. Due to the rare occurrence of tick paralysis cases and the lack of a surveillance system, literature is limited and the exact number of cases in the world cannot be quantified.

**Methods:**

We conducted a meta-analysis of medical literature documenting 121 tick paralysis cases from 1940 to present day using PubMed. Cases were stratified based upon geographic location, tick species, and clinical manifestations.

**Results:**

The compilation includes 71 cases in North America, 14 cases in Australia, 33 cases in Asia, two cases in South America, and one case in Africa. In North America, 36.62% of cases (n=26) were caused by *Dermacentor andersoni* and in Australia, 50% of cases (n=7) were caused by *Ixodes holocyclus*. Dermacentor, Amblyomma, Rhipicephalus, and Hyalomma tick species were the primary culprits in Asia. The ticks involved in the cases in South America and Africa were not identified. Facial palsy was the most commonly reported primary symptom documented in 35 cases, followed by gait ataxia seen in 26 cases. Three cases involved patients with advanced disease causing respiratory depression and cardiopulmonary arrest.

**Table 1:**
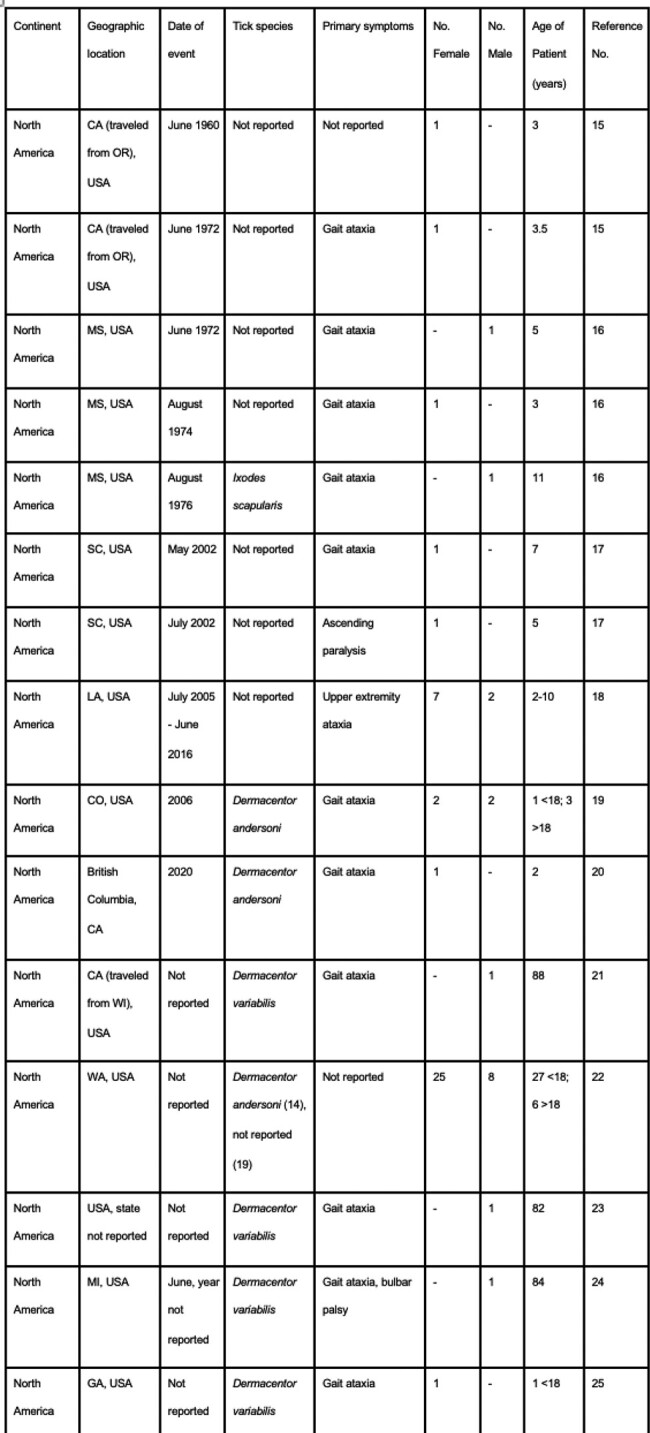
Tick Paralysis Cases Documented 1940-Present

**Table 2:**
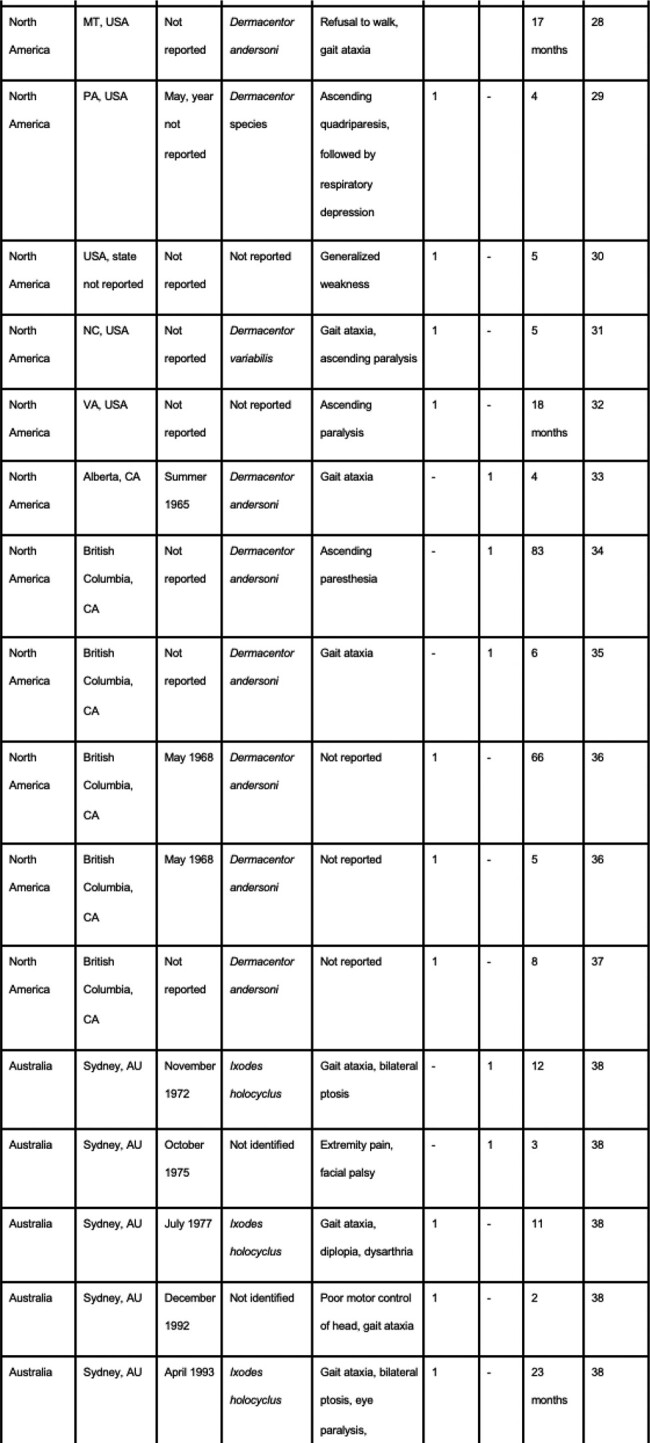
Tick Paralysis Cases Documented 1940-Present

**Table 3:**
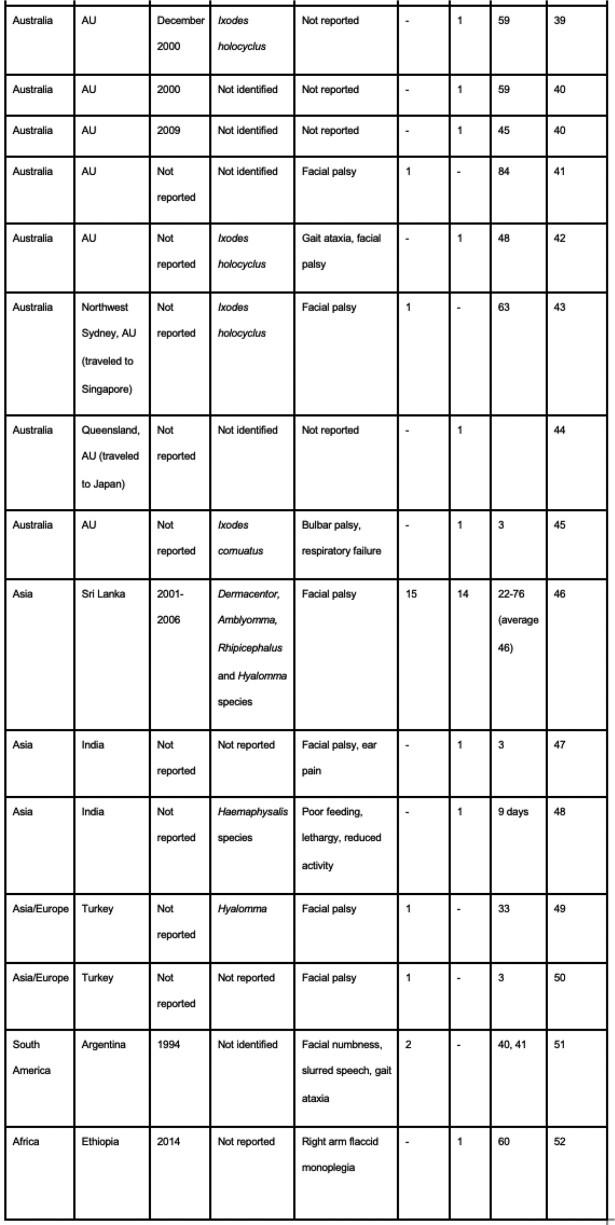
Tick Paralysis Cases Documented 1940-Present

**Conclusion:**

This collection of cases illustrates a wider geographic distribution of tick species than previously documented and suggests the evolving climate and urbanization have significant effects on the expansion of their habitable environments and subsequently, human exposure and illness. Additionally, the meta-analysis supports the need for further education regarding this rare type of tick-borne disease for healthcare providers to ensure prompt diagnosis, appropriate treatment, and improved patient outcomes.

**Disclosures:**

**All Authors**: No reported disclosures

